# The epidemiology of haemodialysis catheter infections in Australia, 2016–20: a prospective cohort study

**DOI:** 10.5694/mja2.70014

**Published:** 2025-07-24

**Authors:** Benjamin Lazarus, Kevan R Polkinghorne, Martin P Gallagher, Jayson Catiwa, Nicholas A Gray, Sarah Coggan, Kathryn R Higgins, Girish Talaulikar, Stephen P McDonald AM, Sradha Kotwal

**Affiliations:** ^1^ The George Institute for Global Health Sydney NSW; ^2^ Monash University Melbourne VIC; ^3^ Centre for Health Services Research the University of Queensland Brisbane QLD; ^4^ Monash Medical Centre Melbourne VIC; ^5^ Prince of Wales Hospital Sydney NSW; ^6^ Sunshine Coast University Hospital Birtinya QLD; ^7^ Canberra Hospital Canberra ACT; ^8^ College of Medicine Australian National University Canberra ACT; ^9^ Australia and New Zealand Dialysis and Transplant (ANZDATA) Registry Adelaide SA; ^10^ University of Adelaide Adelaide SA

**Keywords:** Kidney diseases, Renal dialysis, Vascular diseases, Infection control

## Abstract

**Objectives:**

To investigate the epidemiology in Australia of catheter‐related infections in a national cohort of adults with kidney failure with incident haemodialysis central venous catheters (CVCs).

**Study design:**

Cohort study nested within a cluster‐randomised trial (REDUcing the burden of dialysis Catheter Complications, REDUCCTION); analysis of prospectively collected trial data, linked with Australian and New Zealand Dialysis and Transplant (ANZDATA) registry and state hospitalisations data.

**Setting:**

Thirty‐four health services in Australia (excluding Western Australia) that provide chronic haemodialysis and participated in the REDUCCTION trial.

**Participants:**

Adults (18 years or older) with chronic kidney failure who received incident haemodialysis CVCs during 20 December 2016 – 31 March 2020.

**Main outcome measures:**

Hospitalisation with any haemodialysis CVC infection; haemodialysis CVC‐related bloodstream infections reported during the trial and verified by an independent panel.

**Results:**

Our analysis included 3943 adults with chronic kidney failure; their mean age was 60.4 years (standard deviation, 15.5 years); 1556 were women (39.5%) and 485 were Aboriginal or Torres Strait Islander people (12.3%). Catheter‐related infections were coded for 644 hospitalisations (24.5 per 100 patient‐years; 95% confidence interval [CI], 22.6–26.4 per 100 patient‐years); the incidence was higher among people under 55 years of age (adjusted incidence rate ratio [IRR], 1.55; 95% CI, 1.21–1.98) and those aged 55–70 years (adjusted IRR, 1.34; 95% CI, 1.05–1.70) than among people over 70 years of age. Community‐onset haemodialysis catheter‐related bloodstream infections were responsible for 159 hospitalisations (8.2% of 1938 infection‐related hospitalisations); 57 of 650 infection‐related hospitalisations of people under 55 years of age (8.8%), 62 of 640 of people aged 55–70 years (9.7%), and 40 of 648 of people over 70 years of age (6.2%). The median length of hospital stay with community‐onset haemodialysis CVC‐related bloodstream infections was ten days (interquartile range, 5–15 days), metastatic spread of infection was detected in twelve cases (7.5%), and four people died in hospital (2.5%); 40 removed haemodialysis CVCs did not require replacement. Nineteen of 121 hospitalisations for which the information was available included intensive care unit admissions (15.7%; median stay, 2.7 days; IQR, 1.1–4.6 days). The risk of haemodialysis CVC‐related *Staphylococcus aureus* bloodstream infection declined with age (relative risk ratio, 0.65 per decade; 95% CI, 0.47–0.89).

**Conclusions:**

The health burden of haemodialysis CVC infections in Australia is substantial, particularly among adults under 70 years of age.

**The known**: People with kidney failure often receive life‐sustaining haemodialysis treatment via central venous catheters (CVCs). Infections as complications of the haemodialysis CVC are, however, a major concern.

**The new**: The incidence of haemodialysis CVC infection‐related hospitalisations of adults with kidney failure in Australia is substantial, and it is higher among those under 70 years of age. Forty of 159 CVC‐related bloodstream infections were in people with functional alternative arteriovenous access for haemodialysis.

**The implications**: The incidence of haemodialysis CVC infections needs to be reduced in Australia. Timely removal of CVCs could be one strategy for achieving this aim.

Haemodialysis, the most frequent type of kidney replacement therapy, depends on reliable vascular access.^1^ For 60–80% of patients, maintenance haemodialysis commences with a central venous catheter (CVC);^2^, ^3^ 20–50% of people receiving maintenance haemodialysis have CVCs,^4^ and CVCs are increasingly considered for long term access in people with frailty or older than 80 years.^5^ Haemodialysis can be more rapidly established with a CVC than with an arteriovenous fistulas or a graft, but CVCs are more susceptible to infection‐related complications.^6^

Infections are a problem for people with haemodialysis CVCs,^7^ but the burden of infection attributable to CVCs is unclear. The reported incidence of haemodialysis CVC‐related bloodstream infections ranges between 0.6 and 6.5 episodes per 1000 catheter days,^8^ and the incidence has probably declined over the past three decades.^9^ The risks of *Staphylococcus aureus* bacteraemia^10^ and haemodialysis catheter‐related bloodstream infections^11^ were higher for people under 65 years of age in some studies, but not others.^12^, ^13^ The impact of these infections may also differ between centres and countries. In a case series in a single United States centre, 34% of people with haemodialysis catheter‐related bloodstream infections were admitted to hospital;^14^ in a Western Australian study, 92% of people required hospital admission, but hospitalisation outcomes were not assessed.^15^

We therefore examined the epidemiology of catheter‐related infections in a national cohort of adults with kidney failure with incident haemodialysis CVCs in Australia. We specifically investigated whether people under 70 years of age were more frequently hospitalised with haemodialysis catheter‐related infections, and whether the impact of such hospitalisations was greater in this age group.

## Methods

We undertook a cohort study nested within a cluster‐randomised trial. We analysed prospectively collected data from the national Reducing the burden of dialysis catheter complications (REDUCCTION) study, linked with Australian and New Zealand Dialysis and Transplant Registry (ANZDATA) data and hospitalisations data from all Australian states and territories except Western Australia.

Using prospectively collected REDUCCTION study data, we established a cohort of adults (18 years or older) who received incident CVCs for commencing or continuing chronic haemodialysis in Australia during 20 December 2016 – 31 March 2020 (Supporting Information, part 1).^16^, ^17^ We excluded people not included in the ANZDATA registry (eg, people who received transient haemodialysis for acute kidney injury), whose first trial haemodialysis catheter was inserted for plasma exchange or an undocumented reason, and people enrolled in Western Australia (fewer than 5% of overall cohort; linked hospitalisation data were not available). Included people were followed from the date of insertion of their first CVC until the removal of their last CVC, death, or 31 March 2020, whichever was earliest. Baseline patient, catheter, and service characteristics were based on REDUCCTION data at the date of the first CVC insertion during the trial (Supporting Information, part 2).

### Data linkage

Data for people enrolled in the REDUCCTION study were linked with the ANZDATA registry and hospital admissions datasets from all Australian states and territories except Western Australia using standard identifiers (name, gender, date of birth, medical record number; Supporting Information, part 3). Any hospitalisations commencing from one year prior to the start of the REDUCCTION study period (20 December 2015) to one year after its end (31 March 2021) were identified in the linked data.

### Hospital admissions

Same‐day admissions and admissions for overnight haemodialysis only were excluded. Temporally continuous episodes of care in which patients were transferred to another hospital or to a rehabilitation facility were treated as single hospitalisations, and the principal diagnostic code (International Classification of Diseases, tenth revision, Australian modification; ICD‐10‐AM) for the initial admission was retained for the entire hospitalisation (Supporting Information, part 4). Hospitalisations with any type of catheter‐related infection were defined as those with a relevant ICD‐10‐AM code for either the principal diagnosis or as a secondary code (Supporting Information, table 1). We calculated the proportion of all infection‐related hospital admissions, categorised by principal ICD‐10‐AM diagnostic code (Supporting Information, table 2),^18^, ^19^, ^20^ that were attributed to haemodialysis catheter‐related infections.

Hospitalisations were defined as being caused by community‐onset haemodialysis catheter‐related bloodstream infections if the principal diagnostic code was compatible with a vascular access device infection or sepsis/bacteraemia, and a haemodialysis catheter‐related bloodstream infection event, which was adjudicated and confirmed during the trial, was reported during the three days preceding or the two days following admission (Supporting Information, part 5). During the trial, hospitals were advised to report all possible haemodialysis catheter‐related bloodstream infections; events were confirmed by an independent panel that judged cases using modified Infectious Diseases Society of America (IDSA) criteria^16^ (Supporting Information, part 1).

Length of hospital stay, requirement for intensive care unit (ICU) admission, time in ICU, in‐hospital mortality, and metastatic spread of the infection to distal sites during the same admission were assessed. A literature review established that infective endocarditis, osteomyelitis, septic arthritis, spinal discitis, and epidural abscess are the most frequent metastatic infection types after haemodialysis catheter‐related bloodstream infections;^21^, ^22^, ^23^, ^24^, ^25^ they were defined by compatible secondary diagnostic codes during the hospital admission caused by a haemodialysis catheter‐related bloodstream infection (Supporting Information, table 3). Time from haemodialysis catheter‐related bloodstream infection to CVC removal and whether the CVC required replacement were assessed. Admissions with principal diagnostic codes not compatible with sepsis/bacteraemia or vascular access device‐related infection, and admissions in which confirmed haemodialysis catheter‐related bloodstream infections occurred more than three days before or two days after admission, were deemed to have not been caused by haemodialysis catheter‐related bloodstream infections alone, and were therefore excluded from analyses of hospitalisations attributed to these infections.

### Statistical analysis

We summarise baseline characteristics and hospitalisation outcomes as numbers and proportions, means with standard deviations (SDs), or medians with interquartile ranges (IQRs). Patient age was categorised as under 55 years, 55–70 years, and over 70 years. The incidence of hospitalisations involving haemodialysis CVC infections was estimated by dividing their number by total follow‐up time. Hospital length of stay was calculated from the index admission date. Follow‐up time was calculated as the number of days from first trial CVC insertion until the final CVC was removed, death, or 31 March 2020, whichever was earliest, plus one day (as some people had their only CVC inserted and removed on the same day). Follow‐up time and outcomes for people who moved to an older age category during follow‐up were partitioned by age category.

To assess associations between patient age category and the incidence of hospitalisation with any haemodialysis CVC infection, adjusted incidence rate ratios (IRRs) with 95% confidence intervals (CIs) were estimated using mixed effects negative binomial regression models. The relationship between age at admission with causative organisms was assessed using multivariable multinomial logistic regression; we reported adjusted risk ratios with 95% CIs. The relationship between log‐transformed length of stay for each first hospitalisation with haemodialysis CVC‐related bloodstream infection and causative organisms was assessed using multivariable linear regression (details for analyses: Supporting Information, part 6). Statistical analyses were undertaken in Stata/BE 18.0.

### Ethics approval

The Sydney Local Health District Human Research Ethics Committee approved the study (2019/ETH07707), as did all relevant jurisdictions (Supporting Information, part 7). Our study adhered to the declaration of Helsinki.

## Results

Of the 6248 patients enrolled in the REDUCCTION trial with incident haemodialysis CVCs during 20 December 2016 – 31 March 2020, we excluded 295 people enrolled in Western Australia, 1949 people without chronic kidney failure, and 61 who received their first haemodialysis CVC for plasma exchange or without a listed reason (Box 1). The mean age of the 3943 included people was 60.4 (SD, 15.5) years, 1556 were women (39.5%), and 485 were Aboriginal or Torres Strait Islander people (12.3%). Of the 2519 people who commenced chronic haemodialysis for incident kidney failure with CVCs, the transition was precipitated by acute kidney injury in 836 cases (33.2%; 21.2% of all people receiving incident CVC) (Box 2). In total, 6580 haemodialysis CVCs were inserted during the study period; 5296 were tunnelled (80.5%) and 4712 were in the right internal jugular vein (71.6%). The median number of haemodialysis CVCs per patient was one (IQR, 1–2), the median cumulative duration of use was 200 days (IQR, 93–362 days). The proportions of patients under 55 years of age or aged 55–70 years who were women or Aboriginal and Torres Strait Islander people were larger than for those over 70 years of age, and the proportions who had histories of cardiovascular disease or cancer were smaller (Box 2).

Box 1 **Derivation of study cohort from the Reducing the burden of dialysis catheter complications (REDUCCTION) study cohort, and their transition to haemodialysis with central venous catheters**

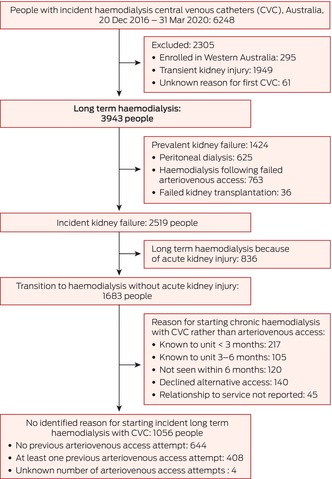

Box 2 Baseline characteristics of 3943 people with incident haemodialysis central venous catheter, by age group*

**Table:** 

Characteristic	All people	Under 55 years	55–70 years	Over 70 years
Number	3943	1335	1381	1227
Age (years), mean (SD)	60.4 (15.5)	42.6 (9.8)	62.9 (4.3)	77.0 (4.9)
Gender (women)	1556 (39.5%)	574 (43.0%)	556 (40.3%)	426 (34.7%)
Ethnic background				
White	2378 (60.3%)	712 (53.3%)	816 (59.1%)	850 (69.3%)
Aboriginal or Torres Strait Islander	485 (12.3%)	282 (21.1%)	172 (12.5%)	31 (2.5%)
Asian	364 (9.2%)	108 (8.1%)	136 (9.8%)	120 (9.8%)
Māori or Pasifika	152 (3.9%)	53 (4.0%)	71 (5.1%)	28 (2.3%)
Other	564 (14.3%)	180 (13.5%)	186 (13.5%)	198 (16.1%)
Private hospital insurance	1029 (26.1%)	254 (19.0%)	374 (27.1%)	401 (32.7%)
Never smoked	1941 (49.2%)	666 (49.9%)	643 (46.6%)	632 (51.5%)
Primary kidney disease				
Diabetic kidney disease	1550 (39.3%)	468 (35.1%)	638 (46.2%)	444 (36.2%)
Glomerular disease	730 (18.5%)	364 (27.3%)	191 (13.8%)	175 (14.3%)
Hypertension	422 (10.7%)	86 (6.4%)	120 (8.7%)	216 (17.6%)
Polycystic kidney disease or reflux nephropathy	272 (6.9%)	122 (9.1%)	98 (7.1%)	52 (4.2%)
Other	912 (23.1%)	272 (20.4%)	312 (22.6%)	328 (26.7%)
Not reported	57 (1.4%)	23 (1.7%)	22 (1.6%)	12 (1.0%)
Immunosuppressant use	513 (13.0%)	228 (17.1%)	176 (12.7%)	109 (8.9%)
Medical conditions				
Coronary artery disease	1164 (29.5%)	218 (16.3%)	439 (31.8%)	507 (41.3%)
Ever had cancer	670 (17.0%)	82 (6.1%)	239 (17.3%)	349 (28.4%)
Peripheral arterial disease	621 (15.7%)	152 (11.4%)	243 (17.6%)	226 (18.4%)
Chronic lung disease	468 (11.9%)	106 (7.9%)	178 (12.9%)	184 (15.0%)
Previous stroke	345 (8.7%)	65 (4.9%)	136 (9.8%)	144 (11.7%)
Indication for first central venous catheter				
Start maintenance haemodialysis	1683 (42.7%)	628 (47.0%)	588 (42.6%)	467 (38.1%)
Acute kidney injury	836 (21.2%)	259 (19.4%)	316 (22.9%)	261 (21.3%)
Arteriovenous access complication	763 (19.4%)	226 (16.9%)	258 (18.7%)	279 (22.7%)
Transfer from peritoneal dialysis	625 (15.9%)	204 (15.3%)	208 (15.1%)	213 (17.4%)
Failed transplant	36 (0.9%)	18 (1.3%)	11 (0.8%)	7 (0.6%)
First catheter tunnelled	3029 (76.8%)	984 (73.7%)	1063 (77.0%)	982 (80.0%)
First catheter in right internal jugular vein	3068 (77.8%)	1049 (78.6%)	1071 (77.6%)	948 (77.3%)
Proceduralist responsible for insertion				
Interventional radiology	2074 (52.6%)	636 (47.6%)	730 (52.9%)	708 (57.7%)
Critical care	627 (15.9%)	205 (15.4%)	221 (16.0%)	201 (16.4%)
Nephrologists	618 (15.7%)	292 (21.9%)	226 (16.4%)	100 (8.1%)
Surgeons	543 (13.8%)	174 (13.0%)	175 (12.7%)	194 (15.8%)
Other	81 (2.1%)	28 (2.1%)	29 (2.1%)	24 (2.0%)
State of enrolment				
New South Wales/Australian Capital Territory	1217 (30.9%)	364 (27.3%)	410 (29.7%)	443 (36.1%)
Victoria or Tasmania	1089 (27.6%)	329 (24.6%)	360 (26.1%)	400 (32.6%)
Queensland	943 (23.9%)	318 (23.8%)	364 (26.4%)	261 (21.3%)
South Australia	398 (10.1%)	135 (10.1%)	157 (11.4%)	106 (8.6%)
Northern Territory	296 (7.5%)	189 (14.2%)	90 (6.5%)	17 (1.4%)
Regional enrolment site	571 (14.5%)	255 (19.1%)	207 (15.0%)	109 (8.9%)
Large service size^†^	1677 (42.5%)	680 (50.9%)	585 (42.4%)	412 (33.6%)

SD = standard deviation.

* At time of enrolment in the REDUCCTION study. Baseline characteristics for people with incident kidney failure (2519 people) or prevalent kidney failure (1424 people) are included in the Supporting Information, table 4; baseline characteristics for people with a central venous catheter that was not precipitated by an acute kidney injury, by age group, are included in the Supporting Information, table 5.

† Large service size: more than 250 patients receiving haemodialysis on 31 December 2016.

### Incidence of hospitalisation with any haemodialysis catheter‐related infection

A total of 10 341 hospitalisations (overnight or longer) and 407 deaths were recorded over 2633.7 patient‐years of follow‐up; multiple‐day hospitalisations were not identified for 168 patients (4.3%). Catheter‐related infections were coded for 644 hospitalisations (24.5 [95% CI, 22.6–26.4] per 100 patient‐years); the incidence was higher for people under 55 years of age (adjusted IRR, 1.55; 95% CI, 1.21–1.98) and for people aged 55–70 years (adjusted IRR, 1.34; 95% CI, 1.05–1.70) than for people over 70 years of age (Box 3). Sensitivity analyses in which hospitalisations with haemodialysis CVC‐related infection was defined by the T82.77 diagnostic code only yielded similar results (Supporting Information, table 6).

Box 3 Hospitalisations with any haemodialysis catheter‐related infection among 3943 people with incident haemodialysis central venous catheters, by age group*

**Table:** 

Characteristic	All people	Under 55 years	55–70 years	Over 70 years
Number of people	3943	1335	1381	1227
Total patient follow‐up time (days)	961 974	302 177	322 099	337 698
Hospitalisations with any haemodialysis central venous catheter infection				
Number	644	235	219	190
Incidence, per 100 patient‐years (95% CI)	24.5 (22.6–26.4)	28.4 (24.8–32.0)	24.8 (21.5–28.1)	20.6 (17.6–23.5)
Adjusted incidence rate ratio (95% CI)^†^	—	1.55 (1.21–1.98)	1.34 (1.05–1.70)	1
Deaths	407 (10.3%)	66 (4.9%)	147 (10.6%)	194 (15.8%)

CI = confidence interval.

* At time of enrolment in the REDUCCTION study.

† Mixed effects negative binomial regression model, adjusted for gender, ethnic background, private hospital insurance, smoking history, indication for the first haemodialysis catheter, immunosuppressant use, primary kidney disease, and atherosclerotic cardiovascular disease.

### Hospitalisations caused by infections

Based on principal ICD‐10‐AM diagnostic codes, 1938 (18.7%) of 10 341 hospitalisations during 20 December 2016 – 31 March 2020 were primarily caused by infections (Box 4), most frequently vascular access device infection (456 hospitalisations), sepsis/bacteraemia (421), pneumonia (375), intra‐abdominal infections (297), cellulitis (138), and bone/joint infections (91 hospitalisations). For 110 of 456 hospitalisations attributed to vascular access device infection (24.1%) and 49 of 421 attributed to sepsis/bacteraemia (11.6%), trial‐confirmed haemodialysis catheter‐related bloodstream infections between three days before and two days after the initial admission were recorded; these 159 hospitalisations comprised 93.5% of the 170 community‐onset haemodialysis catheter‐related bloodstream infections reported and verified during the trial and 8.2% of 1938 infection‐related hospitalisations (Box 4); they accounted for 2181 of 18 913 infection‐related hospitalisations bed‐days (11.5%) (Box 5). Admissions caused by community‐onset haemodialysis catheter‐related bloodstream infections were responsible for 8.8% of all infection‐related hospitalisations of people under 55 years of age (11.7% of bed‐days), 9.7% of those of people aged 55–70 years (13.3% of bed‐days), and 6.2% of those of people over 70 years of age (9.4% of bed‐days) (Box 6).

Haemodialysis catheter‐related bloodstream infection events were also reported during 71 further hospitalisations (Box 4): 27 cases with other principal diagnostic codes, 16 cases in which the infection developed more than two days after admission, and 28 cases in which the infection developed during an admission that precipitated the initial CVC insertion. Eleven haemodialysis catheter‐related bloodstream infections were not associated with hospitalisations. The ICD‐10‐AM diagnostic code T82.77 was recorded for 92 of 159 hospitalisations caused by haemodialysis catheter‐related bloodstream infections (57.9%), 13 of 43 complicated by haemodialysis catheter‐related bloodstream infections (30%), and 254 of 10 139 without trial‐confirmed haemodialysis catheter‐related bloodstream infections (2.5%) (Supporting Information, table 8).

Box 4 All‐cause multiple‐day hospitalisations and confirmed haemodialysis catheter‐related bloodstream infections among 3943 people with incident haemodialysis central venous catheters*

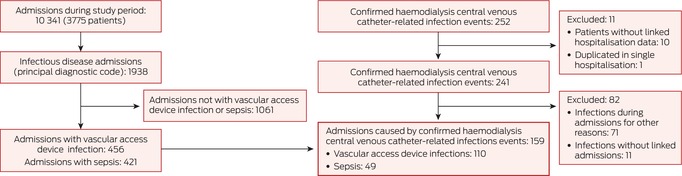

* No linked multiple‐day hospitalisations were identified for 168 people in the study cohort.

Box 5 Hospital admissions caused by infections in people with incident haemodialysis central venous catheters, and associated number of bed‐days*

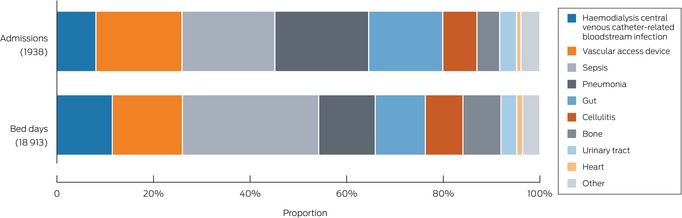

* The data underlying this figure are included in the Supporting Information, table 7.

Box 6 **Proportions of infection‐related hospitalisations and hospital bed‐days attributable to community‐onset haemodialysis catheter‐related bloodstream infections, by age group**

**Table:** 

	Reason for hospital admission	
Age group at admission	Any infection	Haemodialysis catheter‐related bloodstream infection
Hospital admissions	1938	159 (8.2%)
Under 55 years	650	57 (8.8%)
55–70 years	640	62 (9.7%)
Over 70 years	648	40 (6.2%)
Time in hospital (days)	18 913	2181 (11.5%)
Under 55 years	5653	663 (11.7%)
55–70 years	7002	929 (13.3%)
Over 70 years	6258	589 (9.4%)

### Hospitalisations caused by haemodialysis catheter‐related bloodstream infections

The 159 hospitalisations caused by haemodialysis catheter‐related bloodstream infections involved 151 patients; four were hospitalised twice, two were hospitalised three times. *Staphylococcus aureus* was the organism most frequently identified (77 hospitalisations). The median hospitalisation stay was ten (IQR, 5–15) days; metastatic spread of infection was detected in twelve cases (7.5%), including five infective endocarditis, four septic joint, and three osteomyelitis events; four people died in hospital. Nineteen of 121 hospitalisations for which the information was available (15.7%) included ICU admissions (median stay, 2.7 days; IQR, 1.1–4.6 days). The infected CVC was removed within seven days of infection during 140 of 159 admissions (88.1%); 40 removed CVCs (28.6%) were not replaced because a functioning arteriovenous fistula or graft was present (Supporting Information, table 9).

The mean age of 151 patients at the time of their first hospitalisation caused by haemodialysis catheter‐related bloodstream infections was 59.2 (SD, 15.5) years; 114 (75.5%) were under 70 years of age (Box 7). The risk of haemodialysis catheter‐related bloodstream *Staphylococcus aureus* infection hospitalisation declined with age (per decade: adjusted relative risk ratio, 0.65; 95% CI, 0.47–0.89), in contrast to haemodialysis catheter‐related bloodstream infections caused by other *Staphylococcus* spp. and Gram‐negative bacteria (Box 8; Supporting Information, table 10). The mean length of hospital stays caused by *Staphylococcus aureus* (13.5 [95% CI, 7.5–17] days) or *Streptococcus* spp. infections (22.5 [95% CI, 13.5–37] days) was longer than for those caused by other pathogens (other *Staphylococcus* spp.: 8 [95% CI, 4–14] days; Gram‐negative bacteria: 6 [95% CI, 4–9] days; Supporting Information, table 11).

Box 7 Patient characteristics and outcomes of first hospitalisation caused by haemodialysis catheter‐related bloodstream infections, by age group*

**Table:** 

Characteristic	All people	Under 55 years	55–70 years	Over 70 years
Number	151	54	60	37
Age (years), mean (SD)	59.2 (15.5)	42.3 (9.3)	63 (4.6)	77.6 (5.6)
Gender (women)	57 (37.7%)	26 (48%)	20 (33%)	11 (30%)
Ethnic background				
White	103 (68.2%)	34 (63%)	43 (72%)	26 (70%)
Aboriginal or Torres Strait Islander	14 (9.3%)	8 (15%)	6 (10%)	0
Asian	12 (7.9%)	4 (7.4%)	3 (5.0%)	5 (14%)
Māori and Pasifika	2 (1.3%)	1 (1.9%)	1 (1.7%)	0
Other	20 (13.2%)	7 (13%)	7 (12%)	6 (16%)
Private hospital insurance	33 (21.9%)	4 (7.4%)	18 (30%)	11 (30%)
Never smoked	69 (45.7%)	24 (44%)	28 (47%)	17 (46%)
Primary kidney disease				
Diabetic kidney disease	51 (33.8%)	16 (30%)	27 (45%)	8 (22%)
Glomerular disease	34 (22.5%)	19 (35%)	10 (17%)	5 (14%)
Hypertension	23 (15.2%)	5 (9.3%)	8 (13%)	10 (27%)
Polycystic kidney disease or reflux nephropathy	13 (8.6%)	8 (15%)	3 (5.0%)	2 (5.4%)
Other	21 (13.9%)	6 (11%)	9 (15%)	6 (16%)
Not reported	9 (6.0%)	0	3 (5.0%)	6 (16%)
Immunosuppressant use	15 (9.9%)	8 (15%)	5 (8.3%)	2 (5.4%)
Medical conditions				
Ever had cancer	19 (12.6%)	1 (1.9%)	7 (12%)	11 (30%)
Coronary artery disease	50 (33.1%)	12 (22%)	21 (35%)	17 (46%)
Peripheral arterial disease	27 (17.9%)	8 (15%)	10 (17%)	9 (24%)
Previous stroke	11 (7.3%)	3 (5.6%)	4 (6.7%)	4 (11%)
Chronic lung disease	15 (9.9%)	4 (7.4%)	7 (12%)	4 (11%)
Microbiology				
*Staphylococcus aureus*	72 (47.7%)	31 (57%)	29 (48%)	12 (32%)
Other *Staphylococcus* spp. (including *S. epidermidis*)	18 (11.9%)	5 (9.3%)	5 (8.3%)	8 (22%)
*Streptococcus* species (including *Enterococcus* spp.)	12 (7.9%)	4 (7.4%)	5 (8.3%)	3 (8.1%)
Gram‐negative bacteria^†^	35 (23.2%)	8 (15%)	16 (27%)	11 (30%)
Fungal	4 (2.6%)	1 (1.9%)	3 (5.0%)	0
Polymicrobial	10 (6.6%)	5 (9.3%)	2 (3.3%)	3 (8.1%)
Time to catheter removal (days), median (IQR)	2.5 (1.5–3.5)	2 (1.5–3.5)	2.5 (1.5–4.5)	1.5 (1.5–3.5)
Catheter removed within seven days	133 (88.1%)	48 (89%)	53 (88%)	32 (86%)
Catheter removed but not replaced (functioning arteriovenous access)	38 (25.2%)	13 (24%)	19 (32%)	6 (16%)
Length of stay (days), median (IQR)	10 (5–16)	9 (4–15)	11 (5.5–15)	10 (6–18)
Metastatic complications				
Infective endocarditis	5 (3.3%)	2 (3.7%)	3 (5.0%)	0
Osteomyelitis	3 (2.0%)	0	2 (3.3%)	1 (2.7%)
Septic joint	2 (1.3%)	0	1 (1.7%)	1 (2.7%)
Admission to intensive care unit^‡^	19/109 (12.6%)	8/43 (19%)	6/43 (14%)	5/23 (22%)
Intensive care unit stay (days), median (IQR)	2.7 (1.1–4.6)	0.83 (0.1–1.7)	3.6 (1.4–4.6)	3.3 (3.0–3.6)
Died in hospital	4 (2.6%)	1 (1.9%)	3 (5.0%)	0

IQR = interquartile range, SD = standard deviation.

* Four people were hospitalised twice, and two three times with haemodialysis catheter‐related bloodstream infections; in each case, only their first hospitalisation is included here.

† *Escherichia coli*, *Enterobacter cloacae*, *Enterobacter aerogens*, *Klebsiella pneumoniae*, *Klebsiella oxytoca*, *Klebsiella variicola*, *Pseudomonas aeruginosa*, *Pseudomonas luteola*, *Serratia marcescens*, *Serratia liquefaciens*, *Serratia* spp., *Haemophilus influenzae*, *Acinetobacter baumanii* complex, *Citrobacter freundii*, *Achromobacter* spp., *Herbaspirillum huttiense*.

‡ Information not available for all patients.

Box 8 **Estimated probability of specific microorganisms as causes of hospitalisations of 151 people with haemodialysis catheter‐related bloodstream infections**

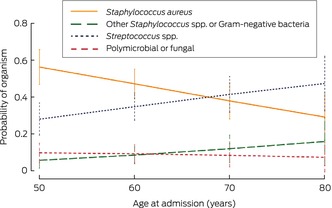

## Discussion

During 2016–20, the incidence of haemodialysis CVC infection‐related hospitalisations among adults with kidney failure in Australia was substantial, and was higher among those under 70 years of age. Of 170 people with community‐onset haemodialysis catheter‐related bloodstream infections, 159 were admitted to hospital (93.5%); these hospitalisations comprised 8.2% of all infection‐related hospitalisations. The median length of hospital stay was ten days (IQR, 5–15 days), metastatic spread of infection was detected in twelve cases (7.5%), and four people died in hospital (2.5%).

The baseline characteristics of people who started incident haemodialysis with CVCs were similar to those recorded in ANZDATA for all people in Australia receiving haemodialysis,^2^ and did not indicate clear contraindications for arteriovenous access. According to vascular access guidelines, age, frailty, and short life expectancy are important factors when considering long term CVCs,^5^ but in our study 69% of incident haemodialysis CVCs were inserted into people under 70 years of age. Interventions that facilitate earlier referral to nephrology services or improve the success rate of arteriovenous access surgery could reduce the need for CVC use, avert infection‐related complications, and improve vascular access outcomes. Genuine engagement and collaborative research with Aboriginal and Torres Strait Islander people are essential for assessing and reducing disparities in their outcomes.^26^

The higher incidence of haemodialysis CVC‐related infections in people under 70 years of age we found is consistent with other reports. People under 65 years of age and adults from ethnic minorities receiving maintenance haemodialysis are more likely than other people to experience *S. aureus* bacteraemia,^10^ which is associated with poorer outcomes; people over 75 years of age less frequently experience bloodstream infections attributable to their haemodialysis CVC.^11^ Although other studies have not found these relationships,^12^, ^13^ it is plausible that the risk of hospitalisation with haemodialysis CVC‐related infections is higher for people under 70 years, as they may be more active and have more contact with others in the community, and their behaviour may place them at greater risk of infection than older people, resulting in more frequent catheter colonisation and infection. People under 60 years have a greater capacity for sweating,^27^ which could favour bacterial growth on moist skin. Further, nasal colonisation with *S. aureus* is more frequent in people under 60 years of age,^28^, ^29^ who may therefore be more susceptible to haemodialysis catheter‐related bloodstream *S. aureus* infections.^23^ Interventions that prevent haemodialysis CVC infections are needed, particularly *S. aureus* infections in younger people.

The characteristics of hospitalisations with confirmed haemodialysis catheter‐related bloodstream infections in Australia differed from those reported in the United States, where fewer than 50% of people with haemodialysis catheter‐related bloodstream infections were hospitalised, and the median length of stay was 4–5 days.^14^, ^23^, ^30^ However, metastatic spread of infection, the need for ICU admission, and in‐hospital mortality were high in both countries. The frequency of haemodialysis catheter‐related bloodstream infections as complications of hospitalisations for other reasons has not previously been reported. As we found that they accounted for 71 of 241 trial‐reported haemodialysis catheter‐related bloodstream infections (29.4%), they should be investigated further. Research into factors involved in differences between countries, such as outpatient use of intravenous antibiotics and preferred choice and route of antimicrobial administration, is needed to assess the relative benefits and risks of different treatment strategies.

The earlier identification and removal of haemodialysis CVCs that are no longer required because alternative functional dialysis access is available could prevent as many as 25% of hospitalisations attributed to haemodialysis catheter‐related bloodstream infections. We found that 40 of 140 CVCs removed within seven days of identified infections did not require replacement because functioning arteriovenous access was available. The incidence of haemodialysis CVC infections in people with alternative dialysis access has not previously been assessed, but delays in removal could result in unnecessary bloodstream infections.^31^ Given natural variation in rates of wound healing and maturation after establishing arteriovenous access, the standard waiting time could be reduced for some patients. Haemodialysis CVC removal practices should be further investigated.

### Limitations

First, we may have underestimated the incidence of haemodialysis CVC‐related infections. As linked hospitalisations were not identified for a small number of people with incident CVCs, we may have missed some hospitalisations with haemodialysis CVC‐related infections. The estimated incidence of hospitalisations attributable to haemodialysis catheter‐related bloodstream infections did not include infections that complicated hospitalisations with other causes, or clinically suspected haemodialysis catheter‐related bloodstream infections that did not satisfy the modified IDSA criteria. Second, the data for this study were collected during a national stepped wedge cluster randomised trial that sought to reduce the incidence of haemodialysis CVC‐related bloodstream infections in Australia. The trial intervention was not effective, but a modest decline in reported infections was found.^9^, ^16^ The estimated burden of infection in our study is therefore a weighted mean of that during the entire trial period, and could be lower than if the trial had not been undertaken. Further studies are needed to better quantify the burden of haemodialysis CVC infections, and to assess whether practice changes, including proceduralist factors, influence the infection risk. Third, the numbers of metastatic infections, ICU admissions, and in‐hospital deaths among people hospitalised with haemodialysis catheter‐related bloodstream infections were too small to assess their frequency by age group. Finally, economic modelling is needed to assess the cost of haemodialysis CVC infections in Australia.^22^, ^30^, ^32^

### Conclusion

The health burden of haemodialysis CVC infections in Australia is substantial, particularly among adults under 70 years of age with incident haemodialysis CVCs. Timely removal of unnecessary haemodialysis CVCs could reduce the number of infections, which would improve the lives of people with kidney failure, particularly those under 70 years of age.

## Open access

Open access publishing facilitated by The University of Queensland, as part of the Wiley – The University of Queensland agreement via the Council of Australian University Librarians.

## Competing interests

No relevant disclosures.

## Data sharing

The individual patient data generated in the trial can be shared in accordance with the trial's data sharing policy and in accordance with the local regulatory and ethics approval for the trial.

## Supporting information


Supplementary methods and results
