# Supplementary material for: The epidemiology of haemodialysis catheter infections in Australia, 2016–20: a prospective cohort study
Source: Med J Aust. 2025 Jul 24;223(5):248–56. doi: 10.5694/mja2.70014 (PMC12399052; doi:10.5694/mja2.70014)
Supplement: Supplementary file 1 — Supplementary methods and results [file MJA2-223-248-s001.pdf]

MJA
The Medical Journal of Australia

Supporting Information

Supplementary methods and results

This appendix was part of the submitted manuscript and has been peer reviewed. It is posted as supplied by the authors.

Appendix to: Lazarus B, Polkinghorne KR, Gallagher MP, et al. The epidemiology of haemodialysis catheter infections in Australia, 2016–20: a prospective cohort study. *Med J Aust* 2025; doi: 10.5694/mja2.70014.

Supplementary methods

1. Details of people enrolled in REDUCCTION

REDUcing the burden of dialysis Catheter Complications (REDUCCTION) enrolled 6248 adult patients who received an incident central venous catheter (CVC) for haemodialysis in 37 Australian nephrology services and examined the effect of a multifaceted CVC care program on the service-wide incidence of haemodialysis catheter-related bloodstream infection (HDCRBSI), using a stepped-wedge cluster randomized design. The 37 participating services were responsible for treating approximately 83% of the people with registered chronic haemodialysis in Australia, as recorded in the Australian and New Zealand Dialysis and Transplant (ANZDATA) registry on 31 December 2016.[1,2] Data linkage with the ANZDATA registry was used to identify patients with chronic kidney failure. Patients enrolled in three Western Australian study sites, who comprised fewer than 5% of the overall cohort, were excluded because linked hospitalisation data were not available in this state. All people with incident CVCs during the REDUCCTION study period were included, and individuals could receive multiple CVCs during the study period. During REDUCCTION, sites were advised to report all possible haemodialysis catheter-related bloodstream infections to a blinded central panel, with deidentified clinical and microbiological data. HDCRBSI events were adjudicated using modified Infectious Diseases Society of America (IDSA) criteria.[3] HDCRBSI was defined as any one of the following: culture of the same organism from both the catheter tip and at least one peripheral percutaneous blood culture, culture of the same organism from at least two blood samples (one from a catheter hub and the other from a peripheral vein), or bacteraemia in the absence of another (non-haemodialysis catheter) source. It was possible for each person with a CVC to have recurrent HDCRBSI, but during the trial each had no more than one confirmed HDCRBSI. The trial was prospectively registered in the Australia and New Zealand clinical trials registry on 23 June 2016 (ACTRN12616000830493).

2. Cohort characteristics

Baseline patient characteristics included age, gender, ethnic background, use of immunosuppressive medication, and diagnosis of diabetes mellitus. Gender was reported by participants categorically as man, woman, or non-binary. Ethnic background was assessed at enrolment in REDUCCTION, categorised as Asian (Chinese, Indian, Malay, Filipino, Vietnamese, or Indonesian), Caucasian, Indigenous Australian (Aboriginal or Torres Strait Islander), Māori, Pacific Islander (Tongan, Samoan, Cook Islander), other, or unknown.[2] Characteristics of each patient's first trial CVC included the indication for insertion as assessed by the treating clinician, venous site, whether it was tunnelled or non-tunnelled, and proceduralist responsible for insertion. Features of the service at which the patient was first enrolled in REDUCCTION included urbanicity (defined as per the original trial, in which services located in an area with a population of more than 100,000 people were considered urban),[2] size (≤ 250 or >250 chronic haemodialysis patients on 31 December 2016), and state (New South Wales/Australian Capital Territory, Victoria/Tasmania, Queensland, Northern Territory, South Australia). Date of death was defined by data collected during REDUCCTION and corroborated by death registry data from each state and territory. Among patients who had their first CVC inserted for commencement of maintenance haemodialysis without a precipitating acute kidney injury, alternative reasons for not having arteriovenous access were recorded, including being new to the unit (<3 months, 3-6 months), previously declining arteriovenous access establishment, or not being seen within the last six months despite being known to the unit. Previous attempts at establishing arteriovenous access were also recorded.

Patient smoking status (ever, never, unknown), primary kidney disease (diabetes mellitus, glomerular disease, hypertension, polycystic disease, reflux nephropathy, other, unknown), and other health conditions (cancer history, coronary artery disease, peripheral arterial disease, cerebrovascular disease, chronic lung disease) were based on ANZDATA registry data when they were recorded prior to or within seven days of first haemodialysis CVC insertion. Patients were considered to have private hospital insurance if it was used in any linked hospital admission during the year preceding the first CVC insertion during the study period. In Australia, access to health care is provided to all citizens and permanent residents, but optional private health insurance is available

at an additional cost. Purchasing private health insurance allows people to choose their treating physician and often facilitates earlier access to elective surgical procedures. People with private hospital insurance can choose whether to use it or not. Private health services may not be available or limited in regional and remote areas, which may influence the decision to purchase it. If a person used public insurance for all admissions, they were considered to not have private insurance. If a patient either did not have any admissions during the preceding year, or all admissions did not have a reported hospital insurance status, they were categorised as having an unknown status of private hospital insurance.

3. Data linkage methods

Probabilistic linkage of REDUCCTION, ANZDATA, and hospitalisations data for New South Wales and the Australian Capital Territory was conducted by the Centre for Health Record Linkage (CHeReL). A master linkage key was constructed using ChoiceMaker software,[4] which used a combination of blocking and scoring strategies to identify exact and possible matches, machine learning techniques, and absolute rules, including an upper and lower probability cutoff of 0.75 and 0.25, to define matches. The false positive rate in a random sample of 1000 manually reviewed patient records in REDUCCTION trial data was 0.5%.

Linkage with Victorian hospitalisation data was conducted by the Centre for Victorian Data Linkage. Deterministic and probabilistic criteria were used. Probabilistic criteria included blocking and weighting based on agreement and disagreement values for other variables. Fuzzy matching for names using the New York State Identification and Intelligence System (NYSIIS) was also used to account for minor differences in the spelling of names. In a random sample of 100 participants, all matches appeared logical.

Similar methods, involving a combination of deterministic and probabilistic linkage, were used in Tasmania (Tasmanian Data Linkage Unit), Queensland (Queensland Linkage Team), and South Australia and the Northern Territory (SA-NT DataLink). In Tasmania, the master linkage map was developed using probabilistic linkage with specialised software after de-duplication of records. Records with at least one mismatch in identifying details were clerically reviewed in an iterative process that also considered additional information, such as previous or alternative names. Additional quality assurance was conducted to ensure that the linked data satisfied a range of logical tests of false positive and false negative matches. All REDUCCTION records for people enrolled at a Tasmanian centre were successfully linked to the Tasmanian hospitalisations dataset. In Queensland, to generate the master linkage file, records from the REDUCCTION cohort were linked deterministically to the hospitalisations data using the medical record number; unlinked records were then matched probabilistically using a program written by the Queensland data linkage team. Nine records from the REDUCCTION source data for people enrolled at a participating Queensland site were not linked to the hospitalisations dataset. In South Australia and the Northern Territory, deterministic linkage was based on exact matches on eleven variables, including medical record number, name, date of birth, and street address. Duplicated records within each dataset were grouped using the same deterministic linkage.

Probabilistic linkage was performed for REDUCCTION records not deterministically matched with hospitalisations data. A variety of linkage variables were used in the probabilistic match, and each linkage variable had a defined weighting related to the specificity of the identifying information. For example, surname and date of birth had more weight than first name or gender. The level of agreement between records was determined by the total weight score for each pair of records. Potential pairs were classified as matches, potential matches, or non-matches. Potential matches were clerically reviewed to determine the final match status.

4. Hospitalisations

Overnight admissions that involved only the provision of overnight haemodialysis were defined by the International Classification of Diseases and Related Health Problems, tenth revision, Australian modification (ICD-10-AM) diagnostic code Z49.1 without any other diagnostic or procedure codes. In NSW, the ACT, Queensland, and Tasmania, up to 50 secondary diagnostic codes were provided for each care episode; in the NT,

Victoria, and SA, each episode respectively contained up to 45, 39, and 29 secondary diagnostic codes. For patients who had their first trial CVC inserted during a hospitalisation, the index hospitalisation was not considered potentially attributable to the CVC. In the case of temporally contiguous hospitalisation encounters, all diagnostic codes in each care episode were considered secondary diagnostic codes for the hospitalisation.

The set of codes (T82.77, T82.7, T80.2, T82.74, T82.75, T82.79, T82.9, T85.7, T85.78) for hospitalisations with any haemodialysis catheter infection was selected after reviewing the literature,[5] the ICD-10-AM codebook, and a manual inspection of codes recorded for patients with confirmed HDCRBSI. In sensitivity analysis, T82.77 ("Infection and inflammatory reaction due to vascular dialysis catheter") alone was used to provide a conservative estimate; this ICD-10-AM diagnostic code is unique to Australia.

Table 1. International Classification of Diseases and Related Health Problems, tenth revision, Australian modification (ICD-10-AM) diagnostic codes used to define hospitalisation with a haemodialysis catheter related infection

ICD-10-AM code	Definition
T82.77	Infection and inflammatory reaction due to vascular dialysis catheter
T82.7	Infection and inflammatory reaction due to cardiac and vascular devices, implants, and grafts, not elsewhere classified
T80.2	Infections following infusion, transfusion, and therapeutic injection
T82.74	Infection and inflammatory reaction due to central vascular catheter
T82.75	Infection and inflammatory reaction due to peripheral vascular catheter
T82.79	Infection and inflammatory reaction due to cardiac and vascular devices, implants, and grafts, not elsewhere classified
T82.9	Unspecified complication of cardiac and vascular prosthetic device, implant, and graft
T85.7	Infection and inflammatory reaction due to other internal prosthetic devices, implants, and grafts
T85.78	Infection and inflammatory reaction due to other internal prosthetic devices, implants, and grafts

Table 2. Principal International Classification of Diseases and Related Health Problems, tenth revision, Australian modification (ICD-10-AM) diagnostic codes used to categorize infectious hospitalisations during the study period

Classification	Principal ICD-10-AM diagnostic code
Vascular access device infection	T80.2 T82.7 T82.74 T82.75 T82.76 T82.77 T82.79 T82.9 T85.7 T85.78
Sepsis or bacteraemia	A02.1 A03.7 A20.7 A21.7 A22.7 A23.7 A24.7 A26.7 A28.01 A28.21 A32.7 A39.7 A40.0 A40.1 A40.2* A40.3 A40.8 A40.9 A41.0 A41.1 A41.2 A41.3 A41.4 A41.5* A41.8 A41.9 A42.7 A54.7 B00.71 B37.7 R65.1 A32.8 A39.2 A39.4 R65.0 A49.0* A49.1 A49.9
Pneumonia	A20.2 A21.2 A31.0 A37* A43.0 A48.1 B01.2 B05.2 B20.6 B25.0 B37.1 B38.0 B38.2 B39.0 B39.2 B44.0 B58.3 B59 B95.3 B96.0 B96.1 J09/J18.9 J20/J22
Cellulitis	A31.1 A36.3 A43.1 A46 A48.0 H00.0 H05.0 H60.0/H60.3 I73.01 J34.0 K12.2 K61* L01.0 L01.1 L02* L03* L05.0 L05.9 L08* M72.6 N73.0 N73.2
Urinary tract infection	N10 N16.0 N39.0 N41.0 N41.1 N41.2
Intra-abdominal	A00* A02.0 A03* A04* A08* A09.0 A09.9 B17.9 D73.3 K35.2 K35.3 K35.8 K36 K37 K38.9 K57.0* K57.12 K57.13 K57.2* K57.32 K57.33 K57.4* K57.52 K57.53 K57.8* K57.92 K57.93 K63.0 K65.0 K67* K75.0 K77.0 K81.0 K81.9 K80.3* T85.71
Bone or joint	M46.2* M46.3* M86* M00* M01*
Endocarditis	A39.5 B33.2 B37.6 I30.1 I32.0 I32.1 I33* I39* I40.0 I41.0/I41.2
Central nervous system infection	A17.0 A20.3 A27.8 A32.1 A39.0 A87* B00.3 B01.0 B02.0 B02.1 B05.1 B06.0 B26.1 B37.5 B38.4 B45.1 B57.4 G00* G01* G02* G03.8 G03.9 G06* G07 G08
Other infections	A* or B* codes not otherwise classified above

The asterisk (*) is a truncation operator and was used to refer to all permutations of diagnostic codes after the initial prefix. For example, M46.2* includes M46.2, M46.20, M46.21, M46.22, and all other codes up to M46.29.

5. Definition of hospitalisation caused by HDCRBSI

Another study[6] applied a time window of HDCRBSI from ten days before to four days after admission to define hospitalisations that were caused by HDCRBSI. We selected a narrower time frame to minimise assumptions about whether there were other reasons for admission unrelated to HDCRBSI that could have also affected hospitalisation outcomes. A time frame limited to two days after admission was selected because health care-associated infections in the general population are defined as those occurring at least 48 hours after admission to a health facility.[7,8] To exclude HDCRBSI caused by care provided during the admission, only admissions in which HDCRBSI were detected within two days of admission were considered to potentially be caused by the HDCRBSI. This was also consistent with a definition of central line-associated bloodstream infection being present on admission if detected from 72 hours before to 72 hours after admission.[9] For one patient, two confirmed HDCRBSI events were identified during the same admission, in two CVCs inserted on consecutive days; we used the date of the first HDCRBSI.

The difference between the number of hospitalisations caused by HDCRBSI using our selected definition and the number of hospitalisations attributable to vascular access device-related infections or sepsis/bacteraemia using diagnostic codes was explained by 1) the absence of a single specific and consistently used diagnostic code for HDCRBSI, 2) the strict criteria used to define HDCRBSI during the REDUCCTION trial, and 3) the requirement for first positive blood culture to be collected between three days before and two days after admission. Given an interest in the outcomes of hospitalisations caused by community-onset HDCRBSI, a conservative case definition was used to minimise bias in hospitalisation outcomes that could be introduced by other concurrent conditions that contributed to the admission.

Table 3. Secondary International Classification of Diseases and Related Health Problems, tenth revision, Australian modification (ICD-10-AM) diagnostic codes used to define metastatic spread of infection during a hospitalisation that was caused by a haemodialysis catheter related bloodstream infection

Site of metastatic spread of infection	ICD-10-AM diagnostic codes
Infective endocarditis	I33 I33.0 I33.9
Osteomyelitis	M46 M46.2* M86.0* M86.1* M86.2* M86.8* M86.9*
Septic arthritis	M00 M00.0* M00.2* M00.9* M01.8*
Spine abscess	G06.0 G06.1 G06.2 M46.3*

The asterisk (*) is a truncation operator and was used to refer to all permutations of diagnostic codes after the initial prefix. For example, M46.2* includes M46.2, M46.20, M46.21, M46.22, and all other codes up to M46.29.

6. Statistical methods

Mixed effects negative binomial regression models. Gender, private hospital insurance status, indication for first CVC, smoking history, primary kidney disease, use of immunosuppressive medications, and presence or absence of atherosclerotic disease (coronary artery disease, peripheral artery disease, or cerebrovascular disease) were modelled as fixed effects. Age was a categorical variable (18-55, 55-70, >70 years old) and updated for patients who transitioned to an older age category during follow-up. Public hospitalisations are funded separately by each state and territory; to adjust for similarities in hospitalisations within a state or territory, the state/territory was included as a random effect. The negative binomial model was used because the mean number of hospitalisations with haemodialysis catheter-related infection was smaller than the variance, and the

Akaike Information Criterion (AIC) and Bayesian Information Criterion (BIC) were smaller than for Poisson models.

Multinomial logistic regression models. The relationship between patient age at time of HDCRBSI admission and the causative organism was assessed in univariable and multivariable multinomial logistic regression models. Organisms that caused HDCRBSI were categorised as *Staphylococcus aureus*, *Streptococcus* spp., other bacteria (*Staphylococcus* spp. or Gram-negative bacteria), and polymicrobial/fungal. HDCRBSI admissions caused by non-aureus *Staphylococcus* spp. or Gram-negative bacteria were defined as the reference outcome category. Gender, insurance status, use of immunosuppressant medications, primary kidney disease, and the presence or absence of atherosclerosis were included in the multivariable model. The estimated probability of each microorganism being the cause of HDCRBSI at different ages was estimated from the model using the margins postestimation command in Stata. For each patient's first hospitalisation caused by HDCRBSI, the association between the causative organism and log-transformed length of stay was assessed using multivariable linear regression, adjusted for age and the same covariates (gender, insurance status, use of immunosuppressant medications, primary kidney disease, presence or absence of atherosclerosis). Geographic and ethnic variations in incident *Staphylococcus aureus* infections and interactions were not assessed.

7. Ethics approval

The relevant jurisdictions for ethical approval included: Sydney Local Health District Human Research Ethics Committee (2019/ETH07707), Australian Capital Territory Human Research Ethics Committee (ETH.7.16.124E), Northern Territory Human Research Ethics Committee (2016-2660), Tasmanian Health and Medical Human Research Ethics Committee (15890), Royal Perth Hospital Human Research Ethics Committee (RGS0000002125), Mater Misericordiae Ltd Human Research Ethics Committee (37691), and University of NSW Human Research Ethics Committee (iRECS5932).

References

1 Australia and New Zealand Dialysis and Transplant Registry. Haemodialysis. In: ANZDATA annual report 2022. https://www.anzdata.org.au/wp-content/uploads/2023/05/Chapter-4-Haemodialysis-ANZDATA-Annual-Report-2022.pdf (viewed Sept 2023).

2 Kotwal S, Coggan S, McDonald S, et al. Reducing the burden of dialysis catheter complications: a national approach (REDUCCTION): design and baseline results. *Kidney360* 2020; 1: 746-754.

3 Kotwal S, Cass A, Coggan S, et al; REDUCCTION Investigators. Multifaceted intervention to reduce haemodialysis catheter related bloodstream infections: REDUCCTION stepped wedge, cluster randomised trial. BMJ 2022; 377: e069634.

4 Borthwick A, Buechi M, Goldberg A. Key concepts in the ChoiceMaker 2 record matching system In: Getoor L, editor. Proceedings of the KDD-2003 Workshop on Data Cleaning, Record Linkage, and Object Consolidation; 2003; Washington DC: Association for Computing Machinery, New York. https://pubs.dbs.uni-leipzig.de/dc/files/Borthwick2003KeyConceptsintheChoiceMaker.pdf (accessed Dec 2024).

5 Johansen KL, Gilbertson DT, Wetmore JB, et al. Catheter-associated bloodstream infections among patients on hemodialysis: progress before and during the COVID-19 pandemic. *Clin J Am Soc Nephrol* 2022; 17: 429-433.

6 Wasik HL, Neu A, Warady B, et al; Standardizing Care to Improve Outcomes in Pediatric End-stage Kidney Disease (SCOPE) Investigators. The cost of hospitalizations for treatment of hemodialysis catheter-associated blood stream infections in children: a retrospective cohort study. *Pediatr Nephrol* 2023; 38: 1915-1923.

7 Australian Commission on Safety and Quality in Health Care. Implementation Guide: Surveillance of Central Line-Associated Bloodstream Infection. Sydney: ACSQHC, 2019. https://www.safetyandquality.gov.au/our-work/infection-prevention-and-control/hai-surveillance/surveillance-central-line-associated-bloodstream-infection (accessed Dec 2023).

8. Haque M, Sartelli M, McKimm J, et al. Health care-associated infections - an overview. Infect Drug Resist 2018; 11: 2321-33.

9. Leeman H, Cosgrove SE, Williams D, et al. Assessing burden of central line-associated bloodstream infections present on hospital admission. *Am J Infect Control* 2020; 48: 216-8.

Supplementary results

Table 4. Baseline characteristics of the study cohort by the indication for the first haemodialysis catheter during the study, by incident kidney failure (2519 people) and prevalent kidney failure (1424 people)

	Incident kidney failure		Prevalent kidney failure			
**Characteristic**	**Start long-term haemodialysis with acute kidney injury**	**Start long-term haemodialysis without acute kidney injury**	**Transfer from peritoneal dialysis**	**Arteriovenous fistula or arteriovenous graft complication**	**Failed transplant**	**Total**
Number of patients	836	1683	625	763	36	3943
Age (years), mean (SD)	59.9 (16.04)	59.2 (15.6)	61.6 (15.3)	62.8 (14.7)	53.8 (14.7)	60.4 (15.5)
Gender (women)	308 (36.8%)	656 (39.0%)	253 (40.5%)	322 (42.2%)	17 (47%)	1556 (39.5%)
Ethnic background						
White	599 (71.7%)	921 (54.7%)	376 (60.2%)	457 (59.9%)	25 (69%)	2378 (60.3%)
Indigenous Australian	58 (6.9%)	287 (17.1%)	33 (5.3%)	105 (13.8%)	2 (5.6%)	485 (12.3%)
Asian	58 (6.9%)	160 (9.5%)	84 (13.4%)	58 (7.6%)	4 (11%)	364 (9.2%)
Māori and Pasifika	23 (2.8%)	67 (4.0%)	32 (5.1%)	30 (3.9%)	0	152 (3.9%)
Other	98 (11.7%)	248 (14.7%)	100 (16.0%)	113 (14.8%)	5 (14%)	564 (14.3%)
Private hospital insurance	253 (30.3%)	379 (22.5%)	181 (29.0%)	208 (27.3%)	8 (22%)	1029 (26.1%)
Never smoked	408 (48.8%)	821 (48.8%)	316 (50.6%)	372 (48.8%)	24 (67%)	1941 (49.2%)
Primary kidney disease						
Diabetic kidney disease	227 (27.2%)	774 (46.0%)	244 (39.0%)	300 (39.3%)	5 (14%)	1,550 (39.3%)
Glomerular disease	160 (19.1%)	270 (16.0%)	144 (23.0%)	144 (18.9%)	12 (33%)	730 (18.5%)
Hypertension	75 (9.0%)	191 (11.3%)	73 (11.7%)	80 (10.5%)	3 (8.3%)	422 (10.7%)
Polycystic kidney disease/reflux nephropathy	30 (3.6%)	98 (5.8%)	58 (9.3%)	78 (10.2%)	8 (22%)	272 (6.9%)
Other/uncertain	327 (39.1%)	318 (18.9%)	104 (16.6%)	155 (20.3%)	8 (22.2%)	912 (23.1%)
Not reported	17 (2.0%)	32 (1.9%)	2 (0.3%)	6 (0.8%)	0	57 (1.4%)
Immunosuppressant use	178 (21.3%)	187 (11.1%)	65 (10.4%)	55 (7.2%)	28 (78%)	513 (13.0%)
Medical conditions						
Ever had cancer	158 (18.9%)	249 (14.8%)	86 (13.8%)	165 (21.6%)	12 (33%)	670 (17.0%)
Coronary artery disease	201 (24.0%)	520 (30.9%)	162 (25.9%)	273 (35.8%)	8 (22%)	1164 (29.5%)

	Incident kidney failure		Prevalent kidney failure			
**Characteristic**	**Start long-term haemodialysis with acute kidney injury**	**Start long-term haemodialysis without acute kidney injury**	**Transfer from peritoneal dialysis**	**Arteriovenous fistula or arteriovenous graft complication**	**Failed transplant**	**Total**
Peripheral arterial disease	101 (12.1%)	277 (16.5%)	83 (13.3%)	159 (20.8%)	1 (2.8%)	621 (15.7%)
Previous stroke	56 (6.7%)	147 (8.7%)	47 (7.5%)	95 (12.5%)	0	345 (8.7%)
Chronic lung disease	100 (12.0%)	202 (12.0%)	54 (8.6%)	109 (14.3%)	3 (8.3%)	468 (11.9%)
First catheter tunnelled)	568 (67.9%)	1378 (81.9%)	562 (89.9%)	648 (84.9%)	33 (92%)	3029 (76.8%)
First catheter in right internal jugular vein	623 (74.5%)	1402 (83.3%)	531 (85.0%)	484 (63.4%)	28 (78%)	3068 (77.8%)
State of enrolment						
New South Wales/Australian Capital Territory	212 (25.4%)	516 (30.7%)	247 (39.5%)	233 (30.5%)	9 (25%)	1217 (30.9%)
Victoria or Tasmania	182 (21.8%)	535 (31.8%)	163 (26.1%)	201 (26.3%)	8 (22%)	1089 (27.6%)
Queensland	281 (33.6%)	299 (17.8%)	164 (26.2%)	190 (24.9%)	9 (25%)	943 (23.9%)
Northern Territory	21 (2.5%)	198 (11.8%)	13 (2.1%)	62 (8.1%)	2 (5.6%)	296 (7.5%)
South Australia	140 (16.7%)	135 (8.0%)	38 (6.1%)	77 (10.1%)	8 (22%)	398 (10.1%)
Regional site	94 (11.2%)	267 (15.9%)	75 (12.0%)	128 (16.8%)	7 (19%)	571 (14.5%)
Large service size*	355 (42.5%)	743 (44.1%)	229 (36.6%)	327 (42.9%)	23 (64%)	1677 (42.5%)

* Large service size defined as a service size of greater than 250 haemodialysis patients on 31 December 2016. SD = standard deviation.

Table 5. Baseline characteristics of patients who started long-term haemodialysis with a central venous catheter that was not precipitated by an acute kidney injury

Characteristic	All people	Under 55 years	55–70 years	Over 70 years
Number	1683	628	588	467
Age (years), mean (SD)	59.2 (15.6)	42.6 (9.6)	62.8 (4.2)	77.0 (4.8)
Gender (women)	656 (39.0%)	262 (41.7%)	239 (40.6%)	155 (33.2%)
Ethnic background				
White	921 (54.7%)	297 (47.3%)	317 (53.9%)	307 (65.7%)
Aboriginal or Torres Strait Islander	287 (17.1%)	181 (28.8%)	95 (16.2%)	11 (2.4%)
Asian	160 (9.5%)	52 (8.3%)	61 (10.4%)	47 (10.1%)
Māori or Pasifika	67 (4.0%)	26 (4.1%)	32 (5.4%)	9 (1.9%)
Other	248 (14.7%)	72 (11.5%)	83 (14.1%)	93 (19.9%)
Private hospital insurance	379 (22.5%)	104 (16.6%)	142 (24.1%)	133 (28.5%)
Relationship with the service				
New (first visit within past three months)	217 (12.9%)	93 (14.8%)	76 (12.9%)	48 (10.3%)
New (first visit within past three to six months)	105 (6.2%)	41 (6.5%)	37 (6.3%)	27 (5.8%)
Known (seen within past six months)*	1056 (62.7%)	352 (56.1%)	377 (64.1%)	327 (70.0%)
Known (not seen within past six months)	120 (7.1%)	67 (10.7%)	33 (5.6%)	20 (4.3%)
Known (declined arteriovenous access)	140 (8.3%)	61 (9.7%)	53 (9.0%)	26 (5.6%)
Unknown	45 (2.7%)	14 (2.2%)	12 (2.0%)	19 (4.1%)

SD = standard deviation.

* Establishment of arteriovenous access had been attempted for 408 people who transitioned to chronic haemodialysis with a central venous catheter without identified explanation (38.6%) and not attempted for 644 people (61.0%); this information was not recorded for four people.

Table 6. Sensitivity analysis in which hospitalisations with haemodialysis catheter infection were defined by T82.77 as the principal or secondary International Classification of Diseases and Related Health Problems, tenth revision, Australian modification (ICD-10-AM) diagnostic code

	Patient age[a]			
**Characteristic**	**<55 years**	**55-70 years**	**>70 years**	**Total**
Number of people	1,335	1,381	1,227	3,943
Total patient follow-up time (days)[b]	302,177	322,099	337,698	961,974
Hospitalisations with T82.77 diagnostic code [b]	134	116	109	359
Number				
Incidence, per 100 patient-years (95% CI)	16.2 (13.5-18.9)	13.2 (10.8-15.5)	11.8 (9.6-14.0)	13.6 (12.2-15.0)
Adjusted incidence rate ratio[c] (95% CI)	1.50 (1.09–2.06)	1.19 (0.87-1.62)	1	—
Deaths	66 (4.9%)	147 (10.6%)	194 (15.8%)	407 (10.3%)

a. Based on age at time of enrolment into the REDUCCTION study.

b. Patients that transitioned to an older age category during follow up, such as from the 55-70 year to the >70-year-old age group, had their follow up time and outcomes partitioned into the appropriate age category.

c. Mixed effects negative binomial regression model, adjusted for gender, ethnicity, private hospital insurance, smoking history, indication for the first HD catheter, immunosuppressant use, primary kidney disease, and atherosclerotic cardiovascular disease.

Table 7. Hospital admissions caused by infections in people with incident haemodialysis central venous catheters, and associated number of bed-days

	Admissions		Bed days	
**Category**	**Number**	**Proportion**	**Number**	**Proportion**
Haemodialysis catheter related bloodstream infection	159	8.2%	2181	11.5%
Vascular access device	346	17.9%	2732	14.4%
Sepsis	372	19.2%	5344	28.3%
Pneumonia	375	19.3%	2208	11.7%
Abdominal	297	15.3%	1970	10.4%
Cellulitis	138	7.1%	1467	7.8%
Bone	91	4.7%	1493	7.9%
Urinary tract	68	3.5%	620	3.3%
Heart	16	0.8%	233	1.2%
Other	76	3.9%	665	3.5%
Total	1938		18913	

Table 8. Proportion of hospitalisations caused by community onset haemodialysis catheter related bloodstream infection (HDCRBSI) that had a compatible diagnostic code for any haemodialysis catheter infection

	Admission caused by HDCRBSI	Admission complicated but not caused by HDCRBSI	Admission without any confirmed HDCRBSI	Total
Hospitalisation with T82.77 diagnostic code in any position	92	13	254	359
Hospitalisation with any haemodialysis catheter infection [a]	145	23	476	644
Total number of admissions	159	43	10139	10341

a. Hospitalisation with any haemodialysis catheter-related infection defined as any of the following ICD-10-AM diagnostic codes in any position: T82.77, T82.7, T80.2, T82.74, T82.75, T82.79, T82.9, T85.7, T85.78.

Table 9. Microbiology and outcomes of 159 admissions attributed to community-onset haemodialysis catheter related bloodstream infections in Australia.

	Total
Number of admissions attributable to community onset haemodialysis catheter related bloodstream infection	159
Microbiology	
Staphylococcus aureus [a]	77 (48%)
Other *Staphylococcus spp.* (including *S. epidermidis*)	19 (12%)
Streptococcus spp. (including *Enterococcus* spp.)	12 (7.5%)
Gram negative bacteria [b]	37 (23%)
Fungal	4 (2.5%)
Polymicrobial	10 (6.3%)
Time to catheter removal (days), median (IQR)	2.5 (1.5–3.5)
Catheter removed within seven days	140 (88%)
Not replaced (functioning arteriovenous access)	40 (25%)
Length of stay (days), median (IQR)	10 (5–15)
Metastatic spread of infection	12 (7.5%)
Infective endocarditis	5 (3.1%)
Osteomyelitis	3 (1.9%)
Septic joint	4 (2.5%)
Admission to intensive care unit [c]	19 (16%)
Length of stay (days), median (IQR)	2.7 (1.1–4.6)
Death in hospital	4 (2.5%)

IQR = interquartile range.

a. Positive blood culture results were available for 158 hospitalisations and the single remaining admission had a principal ICD-10-AM diagnostic code indicating vascular dialysis catheter infection (T82.77) and a second code indicating group A *Streptococcus* sepsis (A40.0).

b. *Escherichia coli*, *Enterobacter cloacae*, *Enterobacter aerogens*, *Klebsiella pneumoniae*, *Klebsiella oxytoca*, *Klebsiella variicola*, *Pseudomonas aeruginosa*, *Pseudomonas luteola*, *Serratia marcescens*, *Serratia liquefaciens*, *Serratia* spp., *Haemophilus influenzae*, *Acinetobacter baumanii* complex, *Citrobacter freundii*, *Achromobacter* spp., *Herbaspirillum huttiense*.

c. Only 121 hospitalisations had data on whether admission to the intensive care unit (ICU) was required or not.

Table 10. Relative risk of haemodialysis catheter related infections hospitalisation caused by different microorganisms by age measured in decades.

Organism category	Relative risk ratio	95% confidence interval
Gram negative bacteria[a] or other *Staphylococcus* species	1	—
Staphylococcus aureus	0.65	0.47-0.89
Streptococcus species	1.20	0.69-2.07
Polymicrobial or fungal organisms	0.74	0.45-1.21

* *Escherichia coli*, *Enterobacter cloacae*, *Enterobacter aerogens*, *Klebsiella pneumoniae*, *Klebsiella oxytoca*, *Klebsiella variicola*, *Pseudomonas aeruginosa*, *Pseudomonas luteola*, *Serratia marcescens*, *Serratia liquefaciens*, *Serratia* spp., *Haemophilus influenzae*, *Acinetobacter baumanii* complex, *Citrobacter freundii*, *Achromobacter* spp., *Herbaspirillum huttiense*.

Table 11. Outcomes of patients' first hospitalisation attributed to haemodialysis catheter related bloodstream infection, by organism

	Staphylococcus aureus	Other *Staphylococcus* spp.	*Streptococcus* spp.	Gram negative bacteria	Fungal	Polymicrobial	Total
Number of patients	72	18	12	35	4	10	151
Age (years), mean (SD)	55.6 (15.1)	62.6 (15.9)	63.3 (14.2)	65.0 (14)	55.6 (17.6)	54.8 (18.1)	59.2 (15.5)
Gender (women)	28 (39%)	5 (28%)	8 (67%)	9 (26%)	2 (50%)	5 (50%)	57 (38%)
Ethnic background							
White	51 (71%)	13 (72%)	4 (33%)	27 (77%)	3 (75%)	5 (50%)	103 (68%)
Indigenous Australian	6 (8%)	1 (6%)	3 (25%)	3 (9%)	0	1 (10%)	14 (9%)
Asian	7 (10%)	0	1 (8%)	4 (11%)	0	0	12 (8%)
Māori or Pasifika	1 (1%)	0	0	0	1 (25%)	0	2 (1%)
Other	8 (11%)	4 (22%)	4 (33%)	1 (3%)	1 (25%)	4 (40%)	22 (15%)
Private hospital insurance	12 (17%)	3 (17%)	2 (17%)	15 (43%)	0	1 (10%)	33 (22%)
Immunosuppressant use	5 (7%)	4 (22%)	1 (8%)	3 (9%)	1 (25%)	1 (10%)	15 (10%)
Length of stay (days), median (IQR)	13.5 (7.5–17)	8 (4–14)	22.5 (13.5–37)	6 (4–9)	7.5 (3.5–10.5)	7.5 (4–10)	10 (5–16)
Metastatic complications [a]							
Infective endocarditis	4 (6%)	0	1 (8%)	0	0	0	5 (3%)
Osteomyelitis	2 (3%)	0	0	1 (3%)	0	0	3 (2%)
Septic joint	1 (1%)	0	1 (8%)	0	0	0	2 (1%)
Admitted to intensive care unit [b]	10 (18%)	1 (8%)	3 (38%)	3 (14%)	0	2 (29%)	19 (17%)
In-hospital mortality [c]	3 (4%)	1 (6%)	0	0	0	0	4 (3%)

a. Among 10 patients for whom metastatic infection complicated their first HDCRBSI admission, 8 occurred among patients under the age of 70 years.

b. Only 109 patients had available data regarding admission to ICU (57 patients with *Staphylococcus aureus*, 12 patients with other *Staphylococcus* spp., 8 patients with *Streptococcus* spp., 21 patients with Gram-negative bacteria, 4 patients with fungal infection and 7 patients with polymicrobial infection)

c. All 4 in-hospital deaths occurred in patients under the age of 70 years.

IQR = interquartile range, SD = standard deviation.
